# Epidemiology of Colorectal Cancer in French Guiana From 2003 to 2017

**DOI:** 10.1002/cam4.71698

**Published:** 2026-03-08

**Authors:** Alolia Aboikoni, Qiannan Wang, Sarah Bailly, Dominique Louvel, Caroline Petorin, Marthe Alogo A. Nwatsok, Paul Ngock Dime, Kinan Drak Alsibai, Mathieu Nacher

**Affiliations:** ^1^ Service d'Hépato‐Gastroentérologie, CHU de Guyane, Site de Cayenne Cayenne French Guiana; ^2^ Registre des Cancers de Guyane (RCan Guyane), Institut Santé des Populations en Amazonie (ISPA) Cayenne French Guiana; ^3^ Service d'Oncologie, CHU de Guyane, Site de Cayenne Cayenne French Guiana; ^4^ Service d'Anatomie Pathologique, CHU de Guyane, Site de Cayenne Cayenne French Guiana; ^5^ Université de Guyane Cayenne French Guiana; ^6^ Centre d'Investigation Clinique (Inserm 1424), Institut Santé des Populations en Amazonie (ISPA) Cayenne French Guiana

**Keywords:** colorectal cancer, french Guiana, incidence, mortality, prognosis

## Abstract

**Background:**

Colorectal cancer (CRC) is a major global public health issue, with 1.9 million new cases and 904,000 deaths in 2022. French Guiana is an overseas territory located in the Amazon region with many unique features, but data on CRC in this area remains limited.

The objective of this work was to evaluate the prognosis of CRC and the associated factors in French Guiana.

**Methods:**

We used the French Guiana Cancer Registry database for the period 2003 to 2017. A survival analysis was conducted. Additionally, standardized incidence and mortality rates were calculated and mapped using QGIS.

**Results:**

During this period, 457 patients were included, with a male predominance (54.7%). The median age was 62 years. One‐third of the population was born abroad. The 5‐year overall survival rate was 47.4% (95% CI [42.1–53.5]). Female sex was associated with better 5‐year survival: 55% [47.2–64.0] vs. 41% [35.0–50.0] for males (*p* = 0.03). In the multivariate analysis, sex and place of birth were independently associated with overall survival (HR = 0.7 [0.5–0.9], *p* = 0.002 for females; HR = 1.5 [1.1–2.1], *p* = 0.005 for those born abroad).

The age‐standardized incidence rate, adjusted to the world population, was 22.6 per 100,000 in men and 17.2 per 100,000 in women. Standardized mortality was 14 per 100,000 for men and 8.5 per 100,000 for women.

**Conclusion:**

In French Guiana, the 5‐year survival rate for CRC was lower than in mainland France. Being born abroad was associated with poorer survival, reflecting health inequalities linked to socio‐economic vulnerability in this territory, despite a lower overall incidence of CRC.

## 1 Background

Colorectal cancer (CRC) is a global public health issue. In 2022, with 1.9 million new cases, it ranked as the third most common cancer globally, and with 904,000 deaths, the second leading cause of cancer‐related mortality [1]. However, the incidence rate varies by region. In general, incidence is higher in developed countries. In South America, there is a progressive increase in both incidence and mortality related to CRC, notably in Brazil [2].

Several risk factors have been reported [3, 4]: Excessive alcohol consumption [5, 6], smoking [7, 8], sedentary lifestyle [9, 10], overweight, obesity [11, 12], a low‐fiber diet [9, 13], high red meat intake [13, 14], and calcium deficiency [14, 15]. Diet is the factor with the highest population attributable fraction. The influence of these exposures and the resources of health systems vary globally, which may affect the epidemiology of this cancer.

In France, CRC is the third most common cancer in men and the second in women. In 2023, 47,582 new cases were reported. In terms of mortality, it ranks second among cancer deaths in men and third in women, accounting for 17,000 deaths annually. The 5‐year survival rate is 63% [16]. To reduce the incidence and mortality of this cancer, an organized screening program was implemented in mainland France in 2008 and extended to overseas territories in 2010.

French Guiana is an overseas department with 286,000 inhabitants as of 2021 [17]. It is located in South America, bordered by Suriname to the west and Brazil to the east. The region is 95% covered by rainforest, with 90%–95% of the population residing along the coast. As a French region, it experiences strong immigration from non‐French‐speaking Latin American and Caribbean countries. In 2015, 30% of the population—and half of the adults—were foreign nationals compared to 6% in mainland France [18, 19]. Other salient features are widespread poverty (50% of the population lives below the poverty line), a high birth rate, and a young population (50% under 25 years of age) [20].

These factors affect almost all major causes of mortality, with foreign‐born populations having worse outcomes for infectious, obstetric, chronic, and certain cancer‐related conditions [21, 22, 23, 24]. A 2024 review highlights the burden of infectious diseases such as HIV and tuberculosis among foreign‐born individuals [21]. Similarly, birthplace is significantly associated with low birth weight, particularly for those from Haiti or Guyana [22]. Regarding breast cancer, women born abroad had worse survival than those born in France, the Antilles, or French Guiana [23].

French Guiana's migratory history and geography have shaped a mix of ethnic and cultural groups with diverse immunogenetic traits, health beliefs, dietary habits, and vulnerabilities. Overweight and obesity are prevalent, with rates double those of mainland France [25]. Some municipalities are remote and only accessible by river or air, which hinders access to healthcare. The health system includes three public hospitals, three local hospitals, and decentralized health centers—now grouped under the CHU de Guyane—as well as three private clinics. Medical staffing is insufficient, especially for specialists [26]. These conditions may cause diagnostic and treatment delays (including in CRC screening), or even renouncement to care.

There is limited data on CRC in French Guiana. From 2010 to 2014, 456 new cancer cases were diagnosed annually, 9% of which were CRC (about 41 cases per year). Standardized incidence and mortality rates were lower than in mainland France: For men, standardized incidence and mortality were 25/100,000 and 6.3/100,000, respectively; for women, 17.4 and 3.8/100,000, respectively [27]. A 2022 study covering the French Antilles and Guiana from 2007 to 2014 reported that CRC accounted for 9% of cancer‐related mortality in men and 11% in women [28].

In this complex context—shaped by ancestral immunogenetic selection pressures, pervasive poverty, distinct dietary patterns, and healthcare access challenges—it is essential to go beyond national statistics and perform a detailed epidemiological analysis of CRC in French Guiana. We hypothesized, as with other cancers, that CRC would exhibit different epidemiological characteristics in terms of survival, incidence, and mortality compared to mainland France, and that these characteristics would vary across Guianese regions.

The primary objective of this study was thus to evaluate the prognosis of colorectal cancer and associated factors. Secondary objectives were to map the age‐standardized incidence and mortality rates by municipality and to report age‐standardized rates relative to the global population. The anticipated benefits of a better understanding of CRC epidemiology include improved prevention, screening, and management in French Guiana, and a reduction in social and territorial health inequalities.

## 2 Methods

### 2.1 Study Design and Data Sources

This was a retrospective, population‐based cohort study conducted using data from the French Guiana Cancer Registry for the period 2003 to 2017. The registry systematically records all new cancer diagnoses among residents of French Guiana since 2003 through multiple mandatory sources, including pathology laboratories, hospital records, discharge summaries, death certificates, and the French National Death Registry. Data are coded using the International Classification of Diseases for Oncology, 3rd version.

Cancer registration in French Guiana is performed within the framework of the French National Cancer Registry Network (FRANCIM), using standardized procedures for case ascertainment, coding, and quality control. Data collection relies on multiple sources, including pathology reports, hospital medical records, and mortality data, with systematic cross‐checking to improve completeness and consistency. Quality indicators routinely used in population‐based cancer registries are monitored in accordance with national and international recommendations. In France, cancer registries are periodically evaluated (approximately every three years) through a national qualification process coordinated by Santé Publique France, the National Cancer Institute (INCa), and Inserm; the French Guiana cancer registry is a certified registry. Taken together, these procedures support the suitability of the registry data for epidemiological analyses, despite challenges related to geographic remoteness and population mobility.

### 2.2 Study Population

We included individuals diagnosed with primary colorectal cancer (using ICD‐10 codes C18.0–C21) between January 1, 2003, and December 31, 2017. Metastatic recurrence of colorectal cancer was an exclusion criterion of the registry. Only residents of French Guiana for at least six months at the time of diagnosis were eligible. To minimize misclassification, duplicate records were cross‐checked across multiple data sources. In addition, cases diagnosed outside French Guiana but residing in the territory were identified through national data linkages. The registry aims for exhaustiveness. We excluded 7 patients diagnosed with appendiceal cancer. In total, 457 cases met the inclusion criteria and were retained for analysis.

### 2.3 Variables and Measurement

Data on demographic, tumoral characteristics, and vital status were extracted from the registry database.

Demographic variables included age at diagnosis, sex, and country of birth. Age was recoded into the following categories: 0–39 years, 40–59 years, 60–79 years, and 80+ years. Places of birth were grouped as France–Europe vs. Foreign. Places of residence were recoded as follows:

‐ Coastal: Iracoubo, Cayenne, Kourou, Matoury, Rémire‐Montjoly, Macouria‐Tonate, Montsinery‐Tonnégrande, Roura, Sinnamary.

‐ Inland: Eastern municipalities (Régina and Saint‐Georges de l'Oyapock) and Western municipalities (Saint‐Laurent du Maroni, Apatou, Maripasoula, Grand‐Santi, Mana, Papaïchton). These are considered remote areas with predominantly rural lifestyles focused on fishing, hunting, and agriculture. Due to small numbers, Saint‐Laurent du Maroni, where the lifestyle is still more urban than in other municipalities in this category, was included in the inland category.

Tumor characteristics variables included cancer topography (caecum, right colon, transverse colon, left colon, sigmoid, rectum), histology, date of biopsy (used as inclusion date), date of last follow‐up, mode of detection (symptoms, organized screening, individual screening, incidental), and vital status.

Vital status at last follow‐up (alive or deceased) was collected through medical records and the French National Death Registry (Fichier National des décès), and subsequently integrated into the French Guiana Cancer Registry.

### 2.4 Primary Endpoint

The primary endpoint was 5‐year overall survival, analyzed globally and by sub‐groups.

### 2.5 Secondary Endpoints

Secondary endpoints were age‐standardized incidence and mortality rates, and geographic mapping of standardized incidence and mortality rates by municipality. Demographic data by age group, sex, and year from 1981 to 2022 were provided by the Guadeloupe Cancer Registry for direct standardization. Municipal‐level demographic data by age group, sex, and year from 2006 to 2017 were provided by INSEE of French Guiana.

### 2.6 Statistical Analysis

#### 2.6.1 Descriptive Analysis

Patient characteristics were described using medians and interquartile ranges for quantitative variables, and counts and percentages for qualitative variables.

#### 2.6.2 Survival Analysis

Overall survival was defined as the time from histological diagnosis to death, from any cause. Patients still alive were censored at the last contact. Person‐time follow‐up, number of events, and incidence density were calculated. Kaplan–Meier estimates were used to determine 5‐year survival. The log‐rank test was used to compare survival curves by sex, age category, birthplace, residence, detection method, tumor site, and histology.

Cox proportional hazards models were used to identify factors associated with survival. Univariate models estimated hazard ratios (HR) and 95% confidence intervals (CI). A multivariate model adjusting for potential confounders was constructed using stepwise forward selection based on the Akaike Information Criterion (AIC). The proportional hazards assumption was tested graphically and using Schoenfeld residuals. Variables violating the assumption (age and histology) were stratified in the final model. All statistical tests were performed under standard conditions with a Type I error risk α = 5%. Analyses were conducted using R version 4.4.0.

#### 2.6.3 Age‐Standardized Incidence Rates

Age‐standardized incidence rates were calculated using direct standardization against the world population. International comparisons were made by averaging annual rates over the study period.

#### 2.6.4 Age‐Standardized Mortality Rates

Age‐standardized mortality rates were also computed using world population standards, and international comparisons were made using the study period averages.

#### 2.6.5 Mapping

Choropleth maps of age‐standardized incidence and mortality rates by municipality were generated using QGIS 3.36. Although current residence may not reflect the location of exposure to risk factors, maps were still produced. Due to data availability, maps were limited to the 2006–2017 period.

### 2.7 Ethical Considerations

The French Guiana Cancer Registry is certified and complies with the Commission Nationale Informatique et des Libertés (CNIL) regulations on data warehouses. CNIL authorization was granted on January 18, 2024 (number DT2230950).

## 3 Results

### 3.1 Population Description

Between January 2003 and December 2017, 457 patients were diagnosed with colorectal cancer. The Table 1 describes the patient characteristics. The population was predominantly male with a median age of 62 years. Most patients lived on the coast, and over two‐thirds were born in France. Cancer was more frequently located on the left side of the lower digestive tract. Organized screening accounted for only 1% of diagnoses. The most common histological type was adenocarcinoma (91.5%).

**TABLE 1:** Population description.

Variable	N (%)
Sex (*n* = 457) male	250 (54.7)
Age *n* = 457	62 (54–73)
Place of birth *n* = 425	
France–Europe	289 (68.0)
Abroad	136 (32.0)
Place of residence *n* = 455	
Coastal	400 (87.9)
Inland	55 (12.1)
Tumor localization *n* = 457	
Caecum, right colon	121 (26.5)
Transverse colon	29 (6.3)
Left colon, sigmoid	157 (34.3)
Rectum	117 (25.6)
Undefined	33 (7.2)
Discovery mode	
Symptoms	300 (65.6)
Individual screening	38 (8.3)
Fortuitous	29 (6.3)
Organized screening program	5 (1.1)
Unspecified	85 (18.6)
Histology *n* = 457	
Colonic adenocarcinoma	418 (91.5)
Others	39 (8.5)

### 3.2 Overall Survival

**FIGURE 1:**
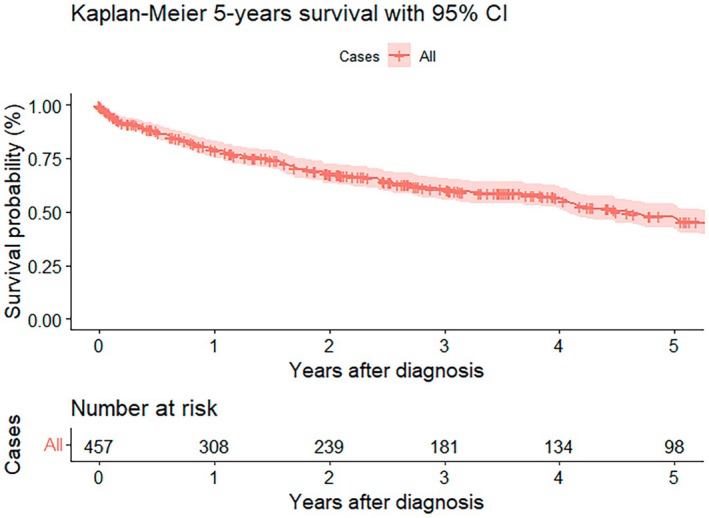
Kaplan–Meier 5‐year survival. Abbreviation: CI, confidence interval.

The 5‐year overall survival probability was 47.4% (95% CI [42.1–53.5]) (Figure 1).

### 3.3 Survival by Demographic Characteristics: Stratified Analysis

**FIGURE 2:**
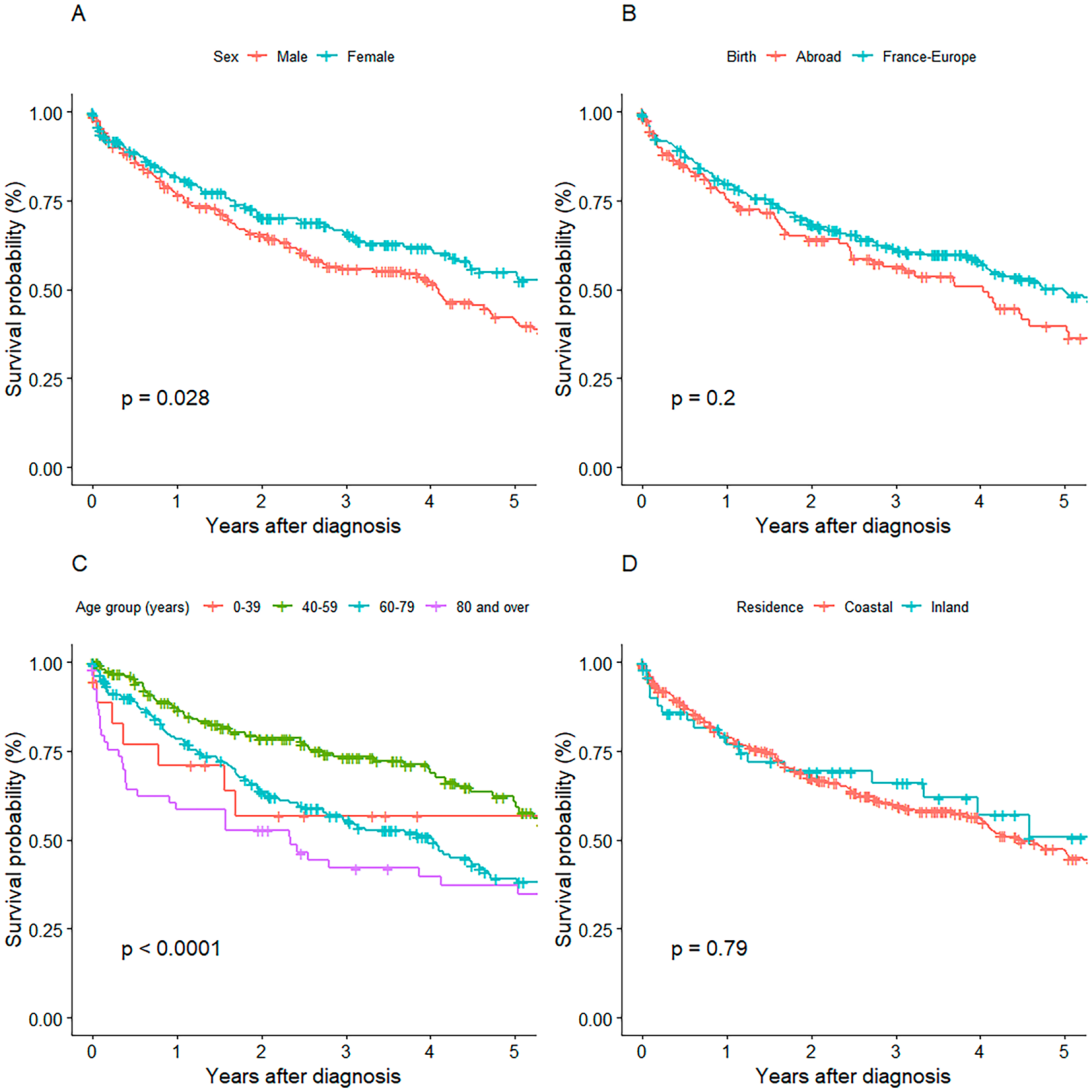
Survival by Demographic Characteristics. (A): Survival by sex. (B): Survival by place of birth. (C): Survival by age group. (D): Survival by place of residence.

Female sex was significantly associated with a higher 5‐year survival rate compared to male sex (*p* = 0.028) (Figure 2A). Although survival curves suggested lower survival for individuals born abroad (Figure 2B), the difference in 5‐year survival by birthplace was not statistically significant (*p* = 0.2). Survival differed significantly by age category; patients aged 40–59 had the best survival, and those over 80 had the poorest (*p* < 0.0001) (Figure 2C). No survival differences were found based on place of residence, coastal and inland (*p* = 0.79) (Figure 2D).

### 3.4 Survival by Tumor Characteristics: Stratified Analysis

**FIGURE 3:**
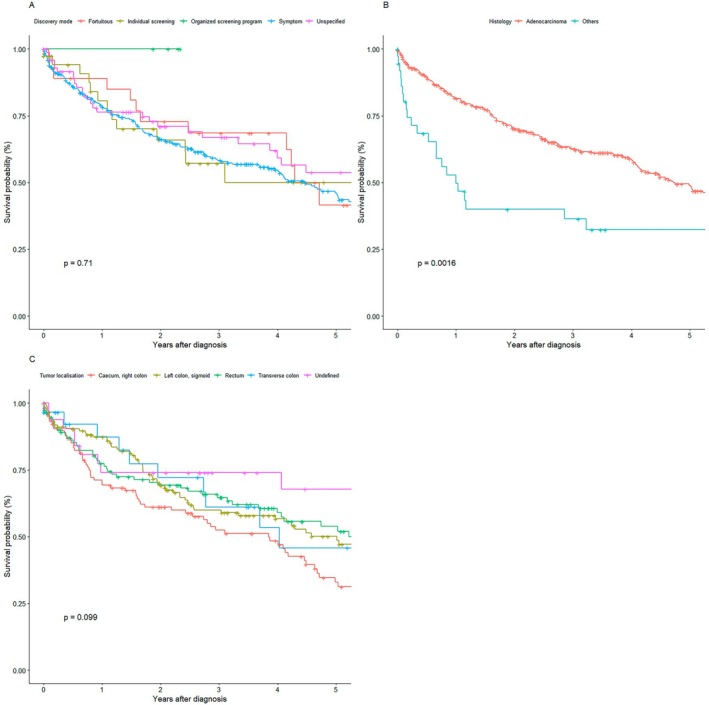
Survival by Tumor Characteristics. (A): Survival by discovery mode. (B): Survival by histology. (C): Survival tumor localization.

There was no significant difference between the different modes of detection (fortuitous, individual screening, organized national screening program, symptomatic patients, and unspecified detection) (*p* = 0.71) (Figure 3A). Regarding the histological type, the adenocarcinoma group was associated with better survival compared to the group “others” (*p* = 0.016) (Figure 3B). Finally, there was no difference in survival with regard to tumor location (caecum, right colon; transverse colon; left colon, sigmoid colon; rectum; *p* = 0.099) (Figure 3C).

### 3.5 Multivariate Analysis of Factors Associated With Overall Survival

In univariate analysis, female sex was significantly associated with better survival than male sex (HR = 0.75, 95% CI [0.58–0.97], *p* = 0.03). Age over 80 (HR = 2.2, 95% CI [0.4–5.0], *p* = 0.04) and non‐adenocarcinoma histology (HR = 1.9, 95% CI [1.3–3.0], *p* = 0.0019) were associated with worse survival. Place of birth, tumor location, and mode of discovery were not significantly associated with overall survival.

The final multivariate Cox model included sex, age, birthplace, and histology. After adjustment and stratification on age and histology, female sex remained independently associated with overall survival (HR = 0.7, 95% CI [0.5–0.9], *p* = 0.002) and place of birth appeared to be significant for survival, with birth abroad being disadvantageous (HR = 1.5, 95% CI [1.1–2.1], *p* = 0.005) (Table 2).

**TABLE 2:** Multivariate Analysis of Factors Associated with Overall Survival.

Variables	Person‐ time	Number of deaths	Incidence	Cox univariate			Cox multivariate		
				HR	IC 95%	*p*	HR	IC 95%	*p*
Total	1,390	243	175						
Sex									
Male	734	146	199	Ref.	Ref.	Ref.	Ref.	Ref.	Ref.
Female	656	97	148	0.75	0.58–0.97	0.03	0.7	0.5–0.9	0.002
Age (years)									
0–39	52	7	134	Ref.	Ref.	Ref.	Stratification		
40–59	544	63	116	0.9	0.4–1.9	0.7		—	—
60–79	629	127	202	1.5	0.7–3.2	0.3			
≥ 80	156	46	295	2.2	0.4–5.0	0.04			
Place of birth									
France–Europe	1,026	172	168	Ref.	Ref.	Ref.	Ref.	Ref.	Ref.
Abroad	343	70	204	1.2	0.9–1.6	0.2	1.5	1.1–2.1	0.005
Place of residence							_	_	_
Coastal	1,262	220	174	Ref.	Ref.	Ref.			
Inland	127	23	181	1.1	0.7–1.6	0.7			
Localization							_	_	_
Caecum, right colon	332	76	229	Ref.	Ref.	Ref.			
Transverse colon	90	12	134	0.6	0.3–1.1	0.09			
Left colon, sigmoid	496	80	161	0.7	0.5–0.9	0.02			
Rectum	338	58	172	0.7	0.5–1.1	0.1			
Undefined	125	17	135	0.6	0.3–1.0	0.06			
Discovery mode							_	_	_
Symptom	979	178	182	Ref.	Ref.	Ref.			
Individual screening	92	16	174	0.9	0.5–1.5	0.8			
Organized screening program	9	0	0	2.9e‐07	0‐inf	1.0			
Fortuitous	86	14	162	0.9	0.5–1.6	0.8			
Unspecified	223	35	157	0.8	0.6–1.3	0.5			
Histology									
Colon Adenocarcinoma	1,310	219	167	Ref.	Ref.	Ref.	Stratification	—	—
Others	71	24	340	1.9	1.3–3.0	0.0019			

### 3.6 Age‐Standardized Incidence

**FIGURE 4:**
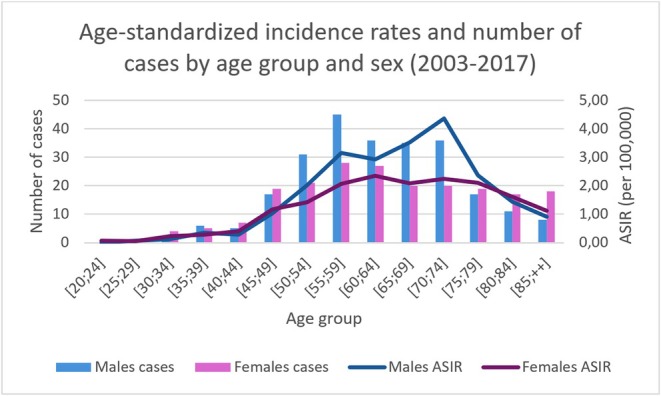
Age‐standardized incidence for 2003–2017. *Abbreviation*: ASIR, Age Standardized Incidence Rate.

The age‐standardized incidence for 2003–2017 was 22.6 per 100,000 in men and 17.2 per 100,000 in women. Incidence increased with age in both sexes and declined after age 80. The highest incidence rates occurred between 55 and 80 years of age for both sexes, with a peak at 70–75 years of age for men (4.4 per 100,000), while for women, there was more of a plateau (around 2 per 100,000) between 55 and 80 years of age (Figure 4).

### 3.7 Age‐Standardized Mortality

**FIGURE 5:**
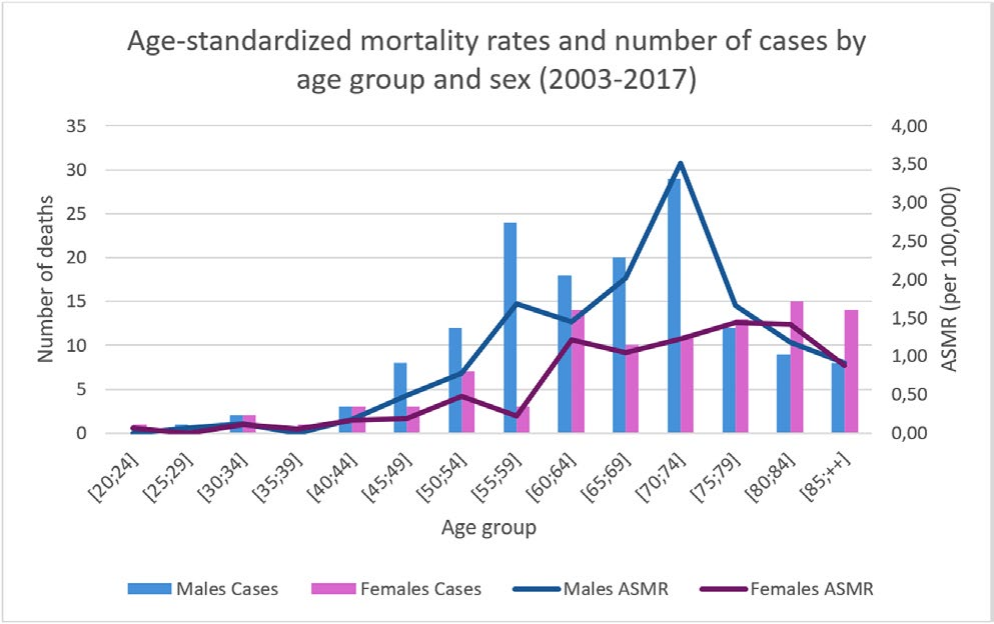
Age‐standardized mortality for 2003–2017. *Abbreviation*: ASMR: Age Standardized Mortality Rate.

The age‐standardized mortality rate was 14 per 100,000 in men and 8.5 per 100,000 in women (2003–2017). Mortality increased in men from the age of 50, peaking at 3.5/100,000 between the ages of 70 and 74. In women, this trend was less pronounced, with a variation between 1 and 1.4/100,000 between the ages of 60 and 85 (Figure 5).

### 3.8 Municipal‐Level Mapping

**FIGURE 6:**
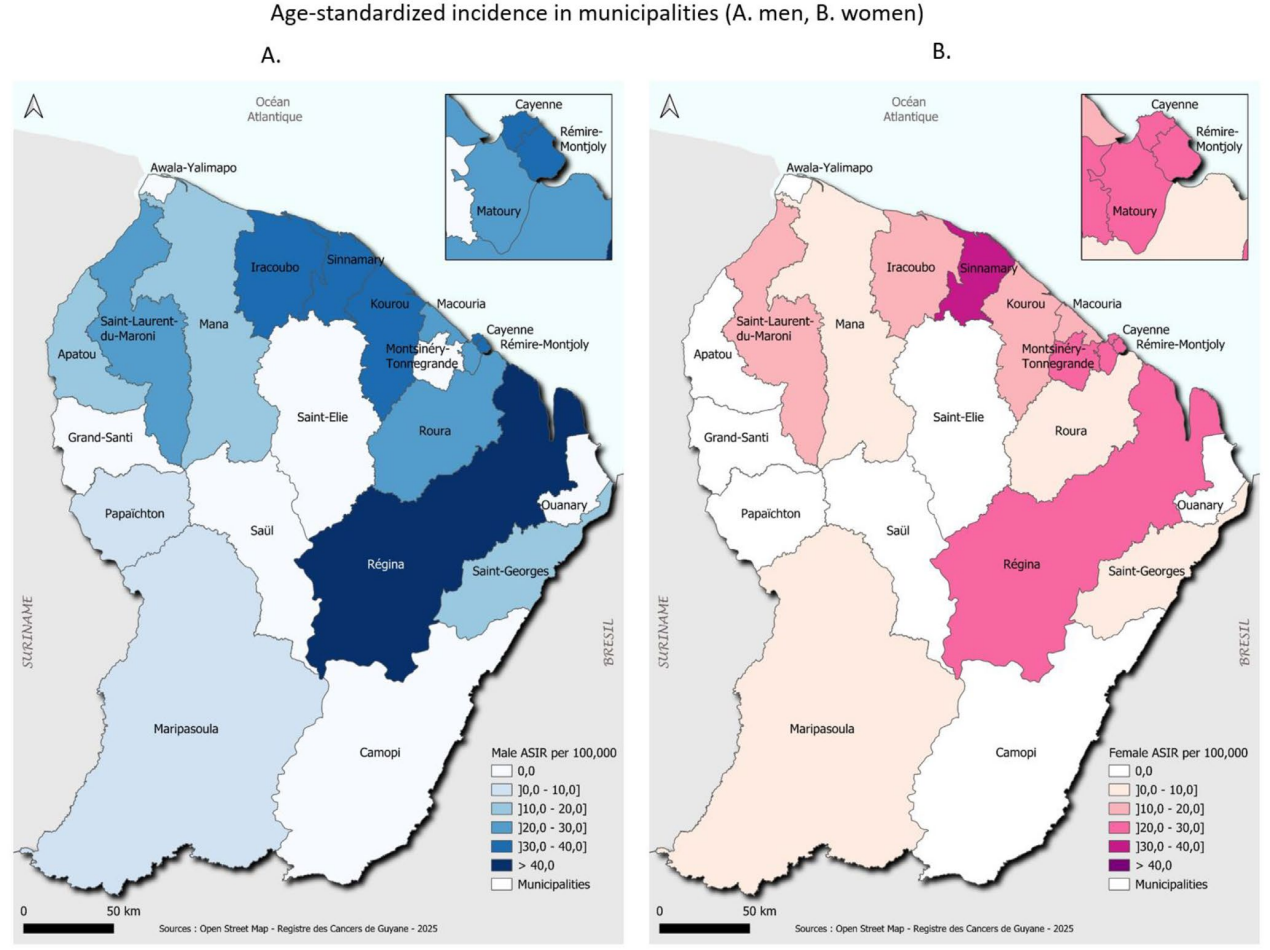
Age‐standardized incidence in municipalities (A. men, B. women). *Abbreviation*: ASIR: Age Standardized Incidence Rate.

Among men, the highest standardized incidence was in Régina (72.8/100,000), followed by Cayenne, Rémire‐Montjoly, Kourou, Sinnamary, and Iracoubo (30–40/100,000) (Figure 6A). Among women, Sinnamary had the highest incidence (31.5/100,000), followed by Régina, Cayenne, Rémire‐Montjoly, Matoury, and Montsinéry‐Tonnégrande (Figure 6B).

**FIGURE 7:**
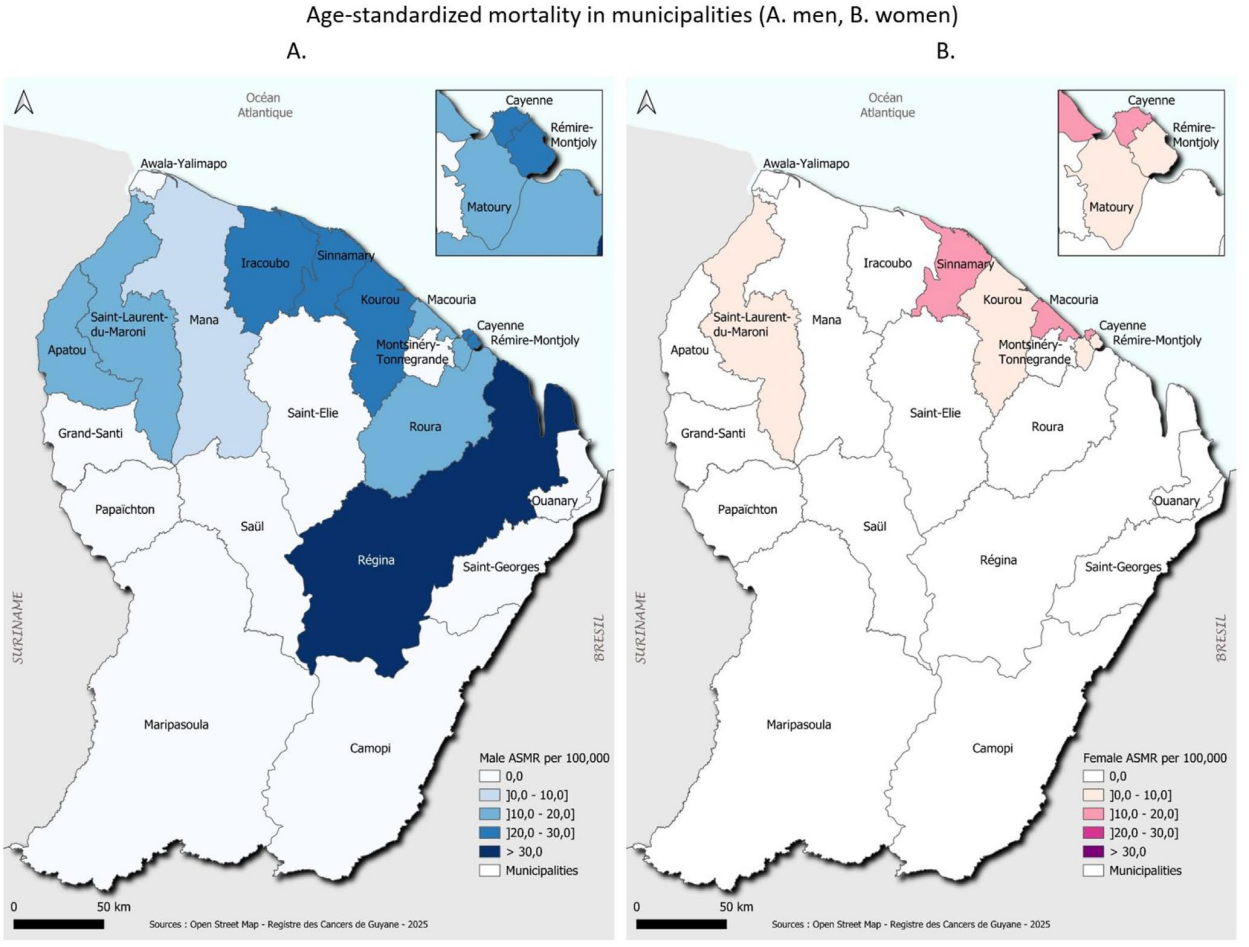
Age‐standardized mortality in municipalities (A. men, B. women). *Abbreviation*: ASMR: Age Standardized Mortality Rate.

For mortality, Régina had the highest standardized rate among men (35.7/100,000), followed by Cayenne, Rémire‐Montjoly, Kourou, Sinnamary, and Iracoubo (Figure 7A). Among women, Macouria‐Tonate, Sinnamary, and Cayenne had the highest rates (17.8, 16.9, and 12.2/100,000, respectively) (Figure 7B).

### 3.9 Regional and International Comparisons

**FIGURE 8:**
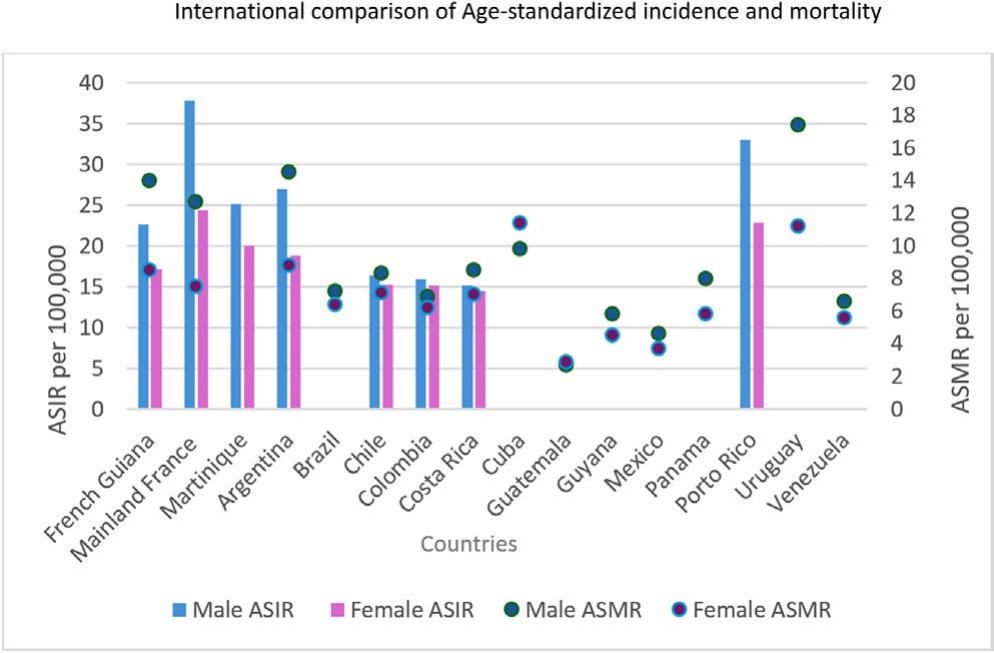
International comparison of Age‐standardized incidence and mortality. ASIR: Age Standardized Incidence Rate. *Abbreviation*: ASMR: Age Standardized Mortality Rate.

Standardized incidence in French Guiana was lower than in mainland France and Martinique. However, standardized mortality was higher in French Guiana than in mainland France (14 vs. 12 per 100,000 in men; 8.5 vs. 7.5 in women). Compared to Latin American countries, female incidence was similar, while male incidence was higher in French Guiana (except in Argentina, where it was higher in both sexes). Overall, mortality in French Guiana was higher (Figure 8).

## 4 Discussion

This study shows that although the age‐standardized incidence rate of colorectal cancer is lower, the age‐standardized mortality rate and overall survival in French Guiana are worse than in mainland France. Men and individuals born abroad had a higher risk of death. These results highlight both the unique epidemiology of CRC in French Guiana and the pervasive territorial and social health inequalities.

The association of older age and male sex with a higher risk aligns with most existing studies [1]. The lower incidence in French Guiana compared to mainland France may be due to cultural dietary differences, such as lower consumption of processed meats and red meat [9, 29] and alcohol [30]. Obesity and sedentary behavior [31]—closely linked to diet—are common in French Guiana (obesity prevalence is double that of mainland France [25]), but genetic factors may also play a role [32]. Although registry data do not allow exploration of genetic influence, the majority of the population in French Guiana has African ancestry. Studies from the U.S. have shown that certain genetic predispositions exist in these populations, affecting both incidence and mortality [32, 33].

However, our findings partially contradict these expectations: While mortality and case‐fatality are increased, incidence is not. A plausible explanation is that local dietary habits reduce exposure to traditional CRC risk factors. Africa is a vast and genetically diverse continent, so predispositions observed in studies in African Americans may not apply to French Guiana.

The relatively high mortality and lower survival, especially among foreign‐born individuals, suggest that massive poverty and limited access to care—especially specialized care—play a significant role. Indeed, the study assumes that being born abroad is a proxy for poverty. While debatable, this proxy is often valid in French Guiana since birth in a poorer foreign country reflects more precarious socioeconomic conditions, language barriers, and difficulties in terms of accessing prevention or care. A study in French Guiana on breast cancer showed a similar link between social inequality (with foreign birth as a proxy) and poor survival [23]. Other studies have found similar results for infectious and obstetric conditions [21, 22]. However, the use of this proxy remains a limitation of this study. Furthermore, CRC screening reduces incidence and improves prognosis, as can be seen in developed countries that have implemented screening programs [34, 35, 36]. However, in French Guiana, participation in screening is very low, at 8% compared to 28.4% in mainland France [37], which could explain the high mortality and lethality rates. In this study, cancers were mainly diagnosed in the context of symptoms, with only 1% diagnosed through organized screening. The small sample size does not allow us to establish with certainty a link between increased mortality and low screening rates, but the question remains. Nevertheless, efforts are being made to promote screening through public awareness campaigns. In addition, the number of specialists in hepatology and gastroenterology has increased over the last three years: There are currently 13 gastroenterologists in the region, compared to two or three previously. Furthermore, since 2025, immunological screening tests have been analyzed in the region at the Cayenne University Hospital laboratory, whereas previously they were sent to mainland France. Finally, oncology services are being restructured. All of these factors should improve screening and treatment for patients.

Compared to Latin American countries, the age‐standardized incidence and mortality rates in French Guiana are higher [1], except when compared to Argentina and Uruguay. Several hypotheses could explain this finding. Firstly, French Guiana is French and, despite structural delays and significant precariousness compared to mainland France, it remains a region with a higher standard of living, westernized, and therefore subject to the risk factors for CRC. Underreporting of cancers in registries could also explain the low incidence and mortality rates in many Latin American countries. In these less favored countries, there are many causes of early mortality, which could impact the epidemiology of CRC [38, 39].

At the municipal level, incidence was generally lower in Eastern and Western municipalities (especially those along the Maroni River), which are more rural and isolated. This observation is consistent with a more rural lifestyle and healthier diets based on fishing and hunting. Surprisingly, the rural municipality of Régina had the highest incidence and mortality among men and the second highest among women. Despite expectations, remote areas did not exhibit higher mortality, suggesting that geographic isolation does not fully explain disparities. However, standardized rates by municipality should be interpreted with caution due to small sample sizes. Indeed, there were a few cases in remote communities in the west and east of the territory. Additionally, residents of remote areas often migrate to the coastal zone, which may lead to underestimation.

This study has limitations but also strengths. The number of patients is relatively small, which can result in random fluctuations. While the registry includes residents of French Guiana, some addresses may be inaccurate, particularly near the border, potentially leading to overestimation. The registry includes limited variables—TNM stage, dietary exposure, family history, and disease history were not available. The stage at diagnosis is a major prognostic factor, but no data were available in the registry to study its impact on survival or on the treatment received. Therefore, we cannot say with certainty how birth abroad is related to a poor prognosis. Although the same observation has been made for many other conditions in French Guiana, we can only speculate that it is because of delayed access to screening and care, and thus a more advanced stage. Future studies should incorporate these variables for a more robust analysis. Finally, we evaluated overall survival, considering all causes of mortality, rather than cancer‐specific survival. Nevertheless, this is a criterion that is widely used in oncology. Despite these limitations, this is the first study of its kind in the region. It provides insights into the CRC situation and suggests areas for improvement. Apparent differences between municipalities and age‐related curves should be interpreted with caution, as small numbers can cause significant noise.

## 5 Conclusion

In conclusion, our initial hypothesis was that colorectal cancer epidemiology in French Guiana differs from that in mainland France. Our findings confirm this, revealing a paradoxical situation: There is a lower age‐standardized incidence rate but a higher age‐standardized mortality rate and poorer 5‐year survival among foreign‐born individuals. These territorial and social health disparities must be addressed. “Outreach” strategies and health mediation could be tested to reduce geographic and social distances in CRC screening and care.

## Author Contributions

Conceptualization: M.N., Q.W., A.A., K.D.A.; Data curation: M.N., A.A., Q.W., K.D.A.; Validation: M.N., A.A.; Formal analysis: M.N., A.A., Q.W.; Writing – original draft preparation: M.N., A.A.; Writing – review and editing: M.N., A.A., Q.W., D.L., S.B., C.P., M.N., P.N.D., K.D.A.; Visualization: M.N., A.A., S.B.; Supervision: M.N., A.A. All authors have read and agreed to the published version of the manuscript.

## Funding

This research was funded by The Agence Régionale de la Santé de Guyane.

## Ethics Statement

The study was conducted in accordance with the Declaration of Helsinki and approved by the Commission Nationale Informatique et Libertés. The French Guiana Cancer Registry is certified and complies with CNIL regulations on data warehouses. CNIL authorization was granted on January 18, 2024 (number DT2230950). Patient consent was waived due to current legislation on registries.

## Conflicts of Interest

The authors declare no conflicts of interest.

## Data Availability

The data that support the findings of this study are available on request from the corresponding author. The data are not publicly available due to privacy or ethical restrictions.
